# Experimental Evaluation of Kinematic Compatibility in Three Upper Limb Exoskeleton Configurations Using Interface Force and Torque

**DOI:** 10.3390/biomimetics11020097

**Published:** 2026-02-01

**Authors:** Hui Zeng, Hao Liu, Longfei Fu, Qiang Cao

**Affiliations:** 1 School of Bailie Mechanical Engineering, Lanzhou City University, No. 572, Anning East Road, Anning District, Lanzhou 730070, China; lzcsxylh@163.com; 2 School of Electrical Engineering, Lanzhou Institute of Technology, No. 1942, East Section of Changjiang Avenue, Lanzhou New Area, Lanzhou 730050, China; flflzgyxy@163.com; 3 School of Mechanical Engineering, Shanghai DianJi University, 300 Shuihua Road, Pudong District, Shanghai 201306, China

**Keywords:** upper limb rehabilitation, exoskeleton robot, kinematic compatibility, human–robot interaction, comparative analysis

## Abstract

Upper limb rehabilitation exoskeletons form a spatial closed kinematic chain with the human arm, where inevitable joint-center and axis misalignment can generate hyperstatic interaction forces and torques. Passive degrees of freedom (DOF) are widely introduced to improve kinematic compatibility, yet different compatible configurations may exhibit distinct wearable performance. This study experimentally compares three compatible four-degree-of-freedom exoskeleton configurations derived from the synthesis of Li et al. using a single reconfigurable rehabilitation robot. The platform is assembled into each configuration through modular passive units and instrumented with two six-axis force–torque sensors at the upper-arm and forearm interfaces. Interaction forces and torques are measured in passive training mode during eating and combing trajectories. For each configuration, tests are performed with passive joints released and with passive joints locked to quantify the effect of passive motion accommodation. Directional and resultant metrics are computed using mean and peak values over movement cycles. Results show that releasing passive joints consistently reduces interaction loading, and Category 2 achieves the lowest forces and torques with the strongest peak suppression, indicating the best practical compatibility.

## 1. Introduction

Stroke and other neurological disorders frequently leave survivors with persistent impairment of upper limb function, which limits independence in daily living [1]. Recovery is strongly associated with intensive, repetitive, and task-relevant practice, yet conventional therapist-delivered training is difficult to scale because it is labor intensive, time constrained, and variable across sessions [2]. Wearable upper limb rehabilitation exoskeletons have therefore attracted sustained interest as a means to deliver repeatable training, to provide quantitative measurement during therapy, and to extend rehabilitation to settings where clinical resources are limited [3].

A defining feature of wearable exoskeletons is the mechanical coupling between the device and the human body through cuffs and attachment interfaces [4,5]. After donning, the exoskeleton and the upper limb form a spatial closed kinematic chain, and coordinated motion is achieved through physical interaction at these interfaces [6]. In an ideal case, the mechanism guides the limb while keeping interaction loads low, so the user experiences assistance rather than constraint. In practice, precise alignment between anatomical joint axes and exoskeleton joint axes is rarely achievable. The shoulder complex exhibits joint-center migration and coupled motion, and the location of attachment interfaces is affected by soft-tissue compliance, cuff placement, and individual anatomy. As the coupled human–robot chain moves, geometric mismatch can introduce redundant constraints that generate hyperstatic forces and torques at the interfaces. These loads vary over the movement cycle and may compromise comfort, safety, and training quality when they become excessive [7,8].

Introducing passive degrees of freedom into the exoskeleton structure is a widely used strategy for mitigating these effects [9]. Passive joints provide motion accommodation paths that allow the mechanism to adjust to joint-center migration and axis misalignment, thereby reducing redundant constraints in the closed chain. Many upper-limb exoskeleton designs incorporate passive joints or passive tracking modules at the shoulder or elbow (EL) and report improved wearing comfort and reduced interaction loading [10,11,12]. However, much of the available evidence is tied to a single device architecture or a local joint module, which makes it difficult to infer general principles for how passive mobility should be allocated across the shoulder and EL subchains.

Configuration synthesis studies provide a more systematic framework by linking compatibility to the kinematic structure of the human–exoskeleton closed chain [13,14]. In particular, Li and coauthors explained incompatibility using mechanism theory and showed that redundant actuation in the closed chain is a primary source of hyperstatic interface loading [15]. Under an upper-limb model that represents the shoulder as a joint with a migrating center of rotation and the EL as a revolute joint, their analysis indicates that eight passive joints should be introduced into a four degree of freedom exoskeleton to avoid hyperstaticity. Importantly, this requirement leads to three families of compatible configurations that differ in how passive degrees of freedom are distributed between the shoulder and EL closed chains [16,17]. This synthesis result is valuable because it frames compatibility as a configuration-level design problem rather than a collection of isolated joint-level adjustments [18].

A practical gap remains between synthesis-level compatibility and wearable performance [19,20,21,22]. Even if multiple configurations satisfy the same theoretical criteria, they may not be equivalent once a real human arm is coupled to the device and moved through functional trajectories [23]. Real systems include finite passive joint ranges, friction, mass distribution, cuff compliance, and subject-specific anatomy, all of which affect how residual mismatch is accommodated and how loads are transmitted to the user [24,25,26]. Moreover, comparisons across different exoskeleton platforms are often confounded by differences in hardware, sensing, and control, which prevents clear attribution of measured interaction forces to configuration differences alone [27,28]. A controlled experimental comparison that holds the hardware platform constant while varying only the passive-joint distribution is therefore needed to translate synthesis insights into configuration selection guidance with engineering value [29].

This study addresses that need by developing a single reconfigurable upper-limb rehabilitation robot that can be assembled into each of the three compatible configuration families using modular passive units. The platform enables the same actuation, mass properties, cuffs, and sensing arrangement to be used across configurations. Human–robot interaction is quantified at both the upper-arm and forearm interfaces using six-axis force–torque sensing, allowing proximal and distal loading to be evaluated separately. Experiments are performed in passive training mode using two representative daily living trajectories, eating and combing, which differ in shoulder excursion and coordination demands. Compatibility is assessed using directional and resultant metrics derived from both mean and peak interaction components, capturing steady loading as well as transient peaks that are most relevant to comfort and safety.

This work makes three contributions. It provides an experimentally controlled framework to compare theoretically compatible configuration families on a common hardware platform. It reports interaction forces and torques at two interfaces under two functionally relevant trajectories, enabling a more complete view of compatibility than single-interface or force-only analyses. It further links the distribution of passive degrees of freedom to measured interaction loading, providing evidence that can guide configuration selection and inform future optimization of passive mobility allocation for safe and comfortable upper-limb rehabilitation.

## 2. Materials and Methods

### 2.1. Three Categories of Rehabilitation Exoskeleton Configurations

When the exoskeleton is attached to the user, the upper limb and the device form a spatial closed kinematic chain [30,31]. Power transmission and coordinated motion during rehabilitation tasks are realized through physical interaction at the connection interfaces between the limb segments and the exoskeleton links [32]. To ensure smooth human exoskeleton interaction and to avoid secondary injury of the affected limb, the rehabilitation system should not generate large constraint forces or large relative motion offsets at these interfaces [33,34,35]. In practice, however, misalignment between the anatomical joint axes of the upper limb and the joint axes of the exoskeleton introduces additional constraints into the closed chain. This misalignment gives rise to hyperstatic forces at the interfaces, and these forces vary with the motion of the chain [36]. As a result, the interaction can become uncomfortable and, in extreme cases, unsafe for the patient [37].

Introducing passive degrees of freedom (DOFs) into the exoskeleton structure is an effective way to compensate for joint axis misalignment [16]. These passive joints allow the kinematic chain of the exoskeleton to adapt to the upper limb, so that the combined human exoskeleton system becomes kinematically compatible and the hyperstatic forces at the connection interfaces are reduced. Based on this design concept, Li et al. [17] proposed a number synthesis approach for exoskeleton configurations that are compatible with the human upper limb. From a mechanism theory point of view, they showed that the incompatibility arises from redundant actuation in the human exoskeleton closed chain. According to the upper limb kinematic model reported in Ref. [38], the shoulder complex can be approximated by a glenohumeral (GH) joint that behaves as a spherical joint with a moving rotational center, and the EL complex can be regarded as a single degree of freedom revolute joint. When the upper limb is modeled as a 4-DOF kinematic chain under this assumption, the results of Li et al. [17] indicate that eight passive joints must be introduced into the exoskeleton chain in order to suppress undesired interaction forces. Furthermore, by distributing these eight passive joints between the shoulder and EL closed chains, Li et al. [17] identified three families of compatible 4-DOF exoskeleton configurations, which are summarized in Table 1. In these three categories, the numbers of passive joints required to achieve closed-chain compatibility at the shoulder and EL joints are denoted by (*f_s_*, *f_e_*), where *f_s_* represents the number of passive DOFs introduced for shoulder closed-chain compatibility and *f_e_* represents the number of passive DOFs introduced for EL closed-chain compatibility. Accordingly, the values of (*f_s_*, *f_e_*) are (3, 5), (4, 4), and (5, 3) for the three categories, respectively, while the total number of passive joints in the human–exoskeleton closed chain remains eight.

On the basis of this analysis, it can be concluded that there are multiple exoskeleton configurations that satisfy the kinematic compatibility conditions with the upper limb. However, not all of these configurations are equally attractive from an engineering point of view [39,40]. To obtain configurations with practical value, the joint distribution patterns of mature upper limb exoskeleton systems, such as Intelli Arm [41], ARMin III [42], and Armeo Power [43], are used as design references. Guided by these systems, the following priorities are adopted for configuration selection. First, the influence of exoskeleton gravity on the active joints and on the upper limb should be minimized as much as possible, by directing the weight of the device toward the base instead of the limb. Second, the passive sub chains between the exoskeleton and the limb should be kept as short as possible in order to maintain efficient transmission of interaction forces and motion. Third, passive joints with a low number of DOF, such as prismatic joints, are preferred because they are structurally simple and mechanically robust [44].

**Figure 1:**
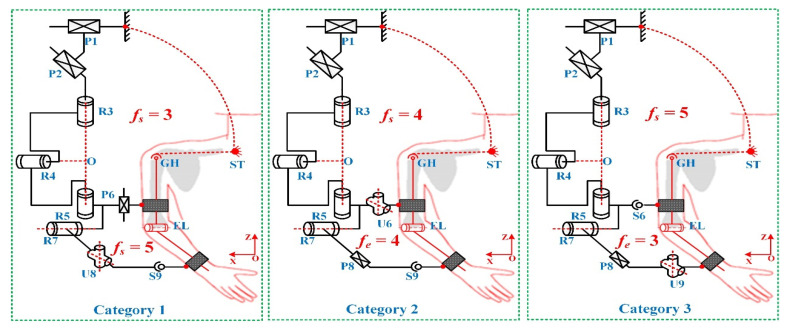
Three representative exoskeleton configurations.

Following these priorities, one representative configuration is selected from each of the three categories proposed by Li et al. [17] for further compatibility analysis and comparison. The three resulting exoskeletons are 4-DOF devices, and their schematic structures are shown in Figure 1. In all three configurations, joints R_3_, R_4_, R_5_, and R_7_ are active joints, which together realize the 3-DOF of the glenohumeral (GH) joint as well as the flexion–extension motion of the EL joint. Specifically, the GH joint enables shoulder adduction/abduction (AD/AB), flexion/extension (FL/EX), and internal/external (IN/EX) rotation of the upper arm.

With respect to the passive joints, the arrangement principles are consistent across the three configurations. Two passive prismatic joints, P_1_ and P_2_, are directly connected to the base and arranged orthogonally in the horizontal plane. Their role is to transfer the weight of the exoskeleton to the base while tracking the horizontal displacement of the GH joint over the workspace, thereby reducing constraint forces transmitted to the user’s limb. All remaining passive joints are located in the connection subchains between the exoskeleton and the upper-limb segments.

The specific type of each passive joint is selected to match the required number of passive degrees of freedom while keeping the connection subchains compact and mechanically robust: a prismatic joint provides one translational DOF, a universal joint provides two rotational DOFs, and a spherical joint provides three rotational DOFs. Importantly, all three configurations are implemented on the same reconfigurable hardware platform using identical passive joint modules (i.e., the same joint models and assembly methods). When switching among configurations, only the number and placement of passive joints are changed. This design choice ensures comparable mechanical characteristics of the passive joints, such as friction, damping, and stiffness, across the three configurations.

Through the introduction and distribution of these passive joints, the human–exoskeleton closed chain is converted into an evenly actuated kinematic system that ensures kinematic compatibility with the upper limb, while keeping the connection subchains short and preserving efficient force and motion transmission. As a result, the subsequent compatibility analysis focuses on the effect of passive DOF distribution at the configuration level, rather than on the optimization of passive joint types. In this work, the three exoskeleton configurations are fixed and used consistently throughout the subsequent compatibility analysis.

### 2.2. Prototype Description of Three Categories Exoskeleton Robot

**Figure 2:**
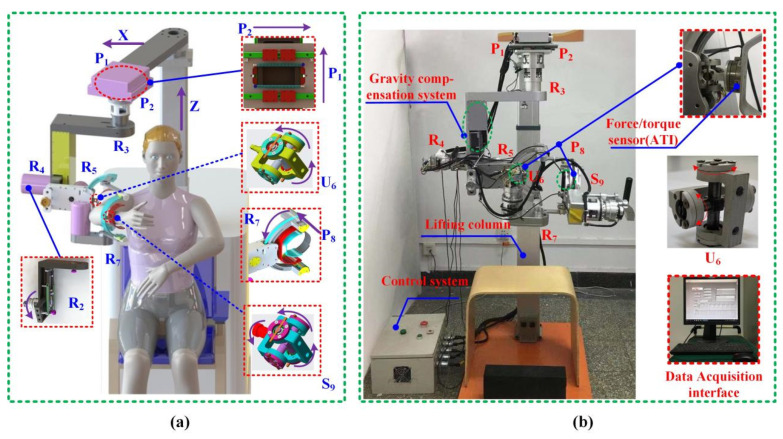
The CAD model of the rehabilitation robot (**a**) and the physical prototype (**b**). Reproduced from Figure 5 in Ref. [45].

Based on the three compatible exoskeleton configurations defined in Section 2.1, a reconfigurable upper limb rehabilitation robot was designed and fabricated. The three-dimensional CAD model and the final prototype are shown in Figure 2. For clarity of hardware description, Figure 2 is reproduced from our previous work (Figure 5 in Ref. [45]), and the source is explicitly acknowledged in the figure caption. The prototype provides a unified mechanical platform that can be assembled into any of the three configurations by replacing the passive modules at the shoulder and EL. This approach allows the kinematic characteristics of the three exoskeleton types to be compared under identical hardware conditions.

The shoulder module contains three active rotational joints, which reproduce FL/EX, AD/AB, and IN/EX rotation of the upper arm. The EL module includes one active joint for FL/EX. All active joints are equipped with dual encoders to ensure accurate joint angle measurements during passive movement. The mechanical frame provides vertical adjustment so that users of different body sizes can be accommodated without altering the kinematic chain. The arm modules are symmetric and can be configured for either the left or the right side, which simplifies clinical operation.

The passive joints in the prototype reflect the structural characteristics extracted from Section 2.1. Two passive prismatic joints, P_1_ and P_2_, are placed between the base and the shoulder module and arranged orthogonally in the horizontal plane. Their function is to follow the floating motion of the GH joint center while transferring the weight of the robot to the base, thereby reducing the load transmitted to the user.

The remaining passive joints are implemented as modular units based on universal and spherical joints. These modules can be mounted or removed without affecting the main structure, allowing the robot to be reconfigured into any of the three compatible exoskeleton types. The choice between universal and spherical joint modules is determined by the required number of passive rotational degrees of freedom at each connection, rather than by differences in joint performance. By adjusting the number and position of these modules, the prototype achieves the desired distribution of passive degrees of freedom while keeping the connection branches short and mechanically efficient.

All passive joint modules are realized using identical joint models and assembly methods, which ensures comparable mechanical characteristics—such as friction, damping, and stiffness—across different configurations. Consequently, the reconfiguration process primarily alters the passive DOF distribution at the configuration level without introducing additional hardware-dependent variability.

The stroke limits of all passive joints were selected based on reported ranges of shoulder joint center migration and anthropometric variability, and were set to exceed the maximum displacement observed during the prescribed tasks, ensuring that no joint reached its mechanical limit during the experiments. Although friction and clearance are unavoidable in practical passive joints and may limit ideal motion accommodation to some extent, their influence remains consistent across all configurations because the same passive joint modules and mechanical structures are used throughout the study. As a result, joint-level frictional effects do not bias the comparative evaluation of configuration-level compatibility.

To measure the human–robot interaction forces during passive rehabilitation movements, six-axis force/torque sensors were installed at the upper arm and forearm interfaces. One Mini45 SI-145-5 sensor (ATI, Apex, NC, USA) was mounted between the upper arm cuff and the robot link, and a second identical sensor was mounted at the forearm interface. Each sensor provides three force components and three moment components, forming the basis for quantitative evaluation of interaction forces and for identifying the influence of different exoskeleton configurations. The performance parameters of the sensors and data acquisition system are summarized in Table 2.

### 2.3. Participants and Experimental Tasks

To evaluate the human robot interaction forces associated with the three compatible exoskeleton configurations defined in Section 2.1, a series of experiments were conducted using the passive training mode of the rehabilitation robot. The robot guided the participants through two representative rehabilitation movements, the eating task and the combing task, while six-dimensional force and torque data were recorded at the upper arm and forearm interfaces. These tasks were selected because they reflect common activities of daily living and involve substantial motion of both the shoulder and EL joints, making them suitable for assessing the interaction characteristics of the three configurations.

Five healthy participants with no history of neurological, musculoskeletal, or cardiopulmonary disorders affecting the upper limbs were recruited. Their anthropometric parameters were measured prior to the experiments, and written informed consent was obtained. Before the main trials, each participant completed introductory sessions to become familiar with the procedure. During all tests, participants were seated comfortably with the trunk supported, and the height of the robot as well as the positions of the arm cuffs were adjusted according to the individual body measurements. The shoulder and EL joints of the participant were aligned as closely as possible with the corresponding robot joints, and the cuffs were tightened sufficiently to maintain stable contact while avoiding excessive compression. All cuffs were fabricated using the same materials and structural design and were reused across all experimental conditions. The wearing tightness was standardized by the same experimenter across subjects to ensure consistent interface conditions. Throughout the experiments, participants were instructed to keep the right arm relaxed.

**Figure 3:**
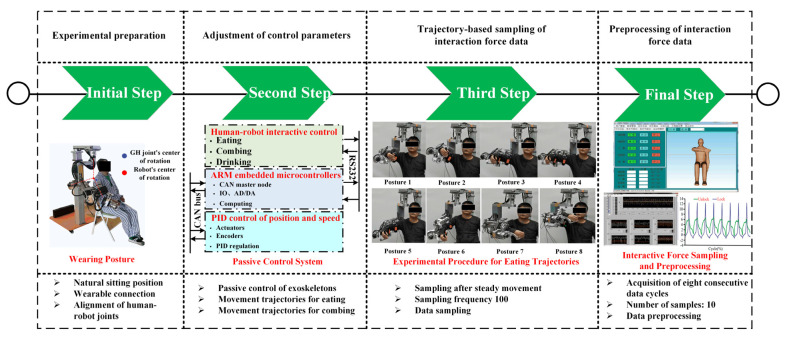
A timeline on the experimental protocol for sampling on human–robot interaction force data. Reproduced from Figure 6 in Ref. [45].

The experimental procedure followed the timeline illustrated in Figure 3. Figure 3 is reproduced from our previous shoulder-focused study (Figure 6 in Ref. [45]) and is cited in the caption. Two experimental modes were tested for each of the three exoskeleton configurations. In the first mode, all passive joints were released, allowing the human–robot closed chain to self-align during movement. In this condition, the passive branch compensated for joint center mismatches, and the interaction forces during the eating and combing tasks were recorded. In the second mode, all passive joints were locked so that no passive motion compensation occurred. This mode allowed the interaction forces to reflect the inherent kinematic mismatch between the upper limb and the exoskeleton. Both modes were carried out under identical task trajectories to permit direct comparison of the influence of passive joint motion on compatibility. Prior to the main experiments, preliminary trials confirmed that none of the passive joints reached their stroke limits during the execution of the eating and combing tasks for any participant.

For every trial, the robot operated in passive mode and followed predefined joint trajectories for the three active shoulder joints and the active EL joint. These trajectories produced smooth transitions from the initial posture to the target posture, with continuous profiles in joint position, velocity, and acceleration. Joint angles measured by the dual encoders were used to ensure accurate tracking of the reference trajectories and to synchronize the kinematic and force measurements. Safety was ensured through mechanical limits, software limits on joint position and velocity, and real-time monitoring of the interaction forces. The motion was stopped immediately if any threshold was exceeded or if the participant reported discomfort.

Interaction forces and torques were recorded using two six-axis sensors mounted at the upper arm and forearm interfaces. The sampling frequency was set to 100 Hz to obtain detailed measurements of the transmitted forces and torques throughout the passive movements. Data collection began only after the robot reached a stable closed-chain state, avoiding transient effects that would otherwise influence the measurements. Although soft tissue deformation and micro-slippage at the cuff–limb interface cannot be completely eliminated in wearable experiments, these effects were consistent across configurations because the same cuffs, attachment locations, and wearing procedures were used throughout the study. Each participant completed ten repetitions of the eating and combing tasks in each experimental mode for each exoskeleton configuration. Every repetition contained eight continuous cycles of the predefined trajectory. All collected data were processed using consistent filtering and selection criteria to ensure reliable comparison across configurations and between the two experimental modes.

This procedure resulted in a complete dataset of interaction forces and torques for all three exoskeleton configurations under both the self-aligned and alignment-free conditions. Accordingly, the present experiments emphasize relative comparisons of interaction forces and torques among configurations under identical interface conditions, rather than absolute force transmission characteristics at the cuff–limb interface. The dataset provides the basis for assessing the kinematic compatibility of each configuration and for understanding how the distribution of passive joints influences the human robot interaction.

### 2.4. Quantitative Metrics for Compatibility

To evaluate the compatibility of the three exoskeleton configurations described in Section 2.1, the interaction forces collected in Section 2.3 were used to construct quantitative metrics. These metrics reflect the mechanical coupling between the upper limb and the device during passive movements and allow direct comparison of the three configurations under identical motion conditions. The interaction forces and torques were analyzed along three orthogonal directions, denoted as the *X*, *Y* and *Z* axes of the local sensor frame.

For each movement cycle of the eating and combing tasks, the three force components *F_x_*, *F_y_*, *F_z_* and the three torque components *T_x_*, *T_y_*, *T_z_* were extracted from the six-axis sensors. Two sets of directional indicators were computed. The first set consisted of the mean value and the peak value of each directional force and torque, calculated over all sampled data in a cycle. If *i* represents one direction of interest, the mean and peak values were obtained from

(1) $${\bar{F}}_{i}=\frac{1}{n}\sum_{k=1}^{n}F_{i}(k),\;F_{i}^{\mathrm{peak}}=\max\left|F_{i}\left(k\right)\right|,$$

and

(2) $${\bar{T}}_{i}=\frac{1}{n}\sum_{k=1}^{n}T_{i}(k),\;T_{i}^{\mathrm{peak}}=\max\left|T_{i}\left(k\right)\right|,$$

where *n* is the number of samples in a cycle. These values characterize the steady interaction level and the instantaneous loading experienced by the participant.

To obtain an overall measure of the interaction loading, total force and total torque were computed as the Euclidean norms of the directional components,

(3) $$F_{\mathrm{tot}}=\sqrt{F_{x}^{2}+F_{y}^{2}+F_{z}^{2}},\;T_{\mathrm{tot}}=\sqrt{T_{x}^{2}+{\;T}_{y}^{2}+{\;T}_{z}^{2}}\;$$

The mean and peak values of these totals were then calculated in the same manner as above. These norms provide a compact representation of the combined effect of the directional forces and torques and allow the three configurations to be compared through a single scalar quantity.

All metrics were computed for the upper-arm and forearm sensors independently. By substituting the interaction forces measured in Section 2.3 into the above definitions, the compatibility of the three exoskeleton configurations can be quantitatively assessed. Configurations producing smaller directional forces, smaller total interaction forces, and reduced peak values were considered to exhibit better mechanical compatibility with the upper limb. These metrics form the basis for the comparisons presented in the results section.

## 3. Analysis and Results

### 3.1. Compatibility Comparison Among Three Configurations

**Figure 4:**
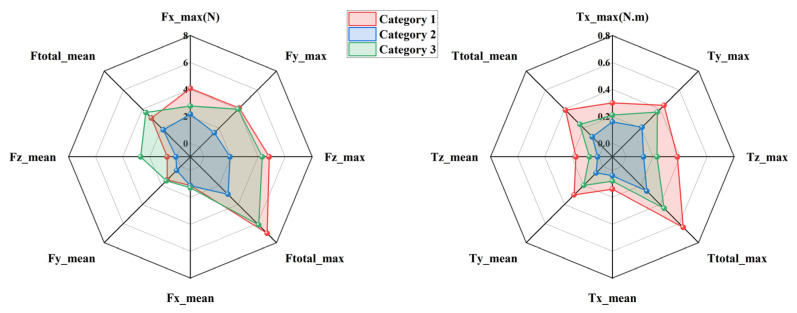
Interaction forces and torques at the forearm during the eating task.

**Figure 5:**
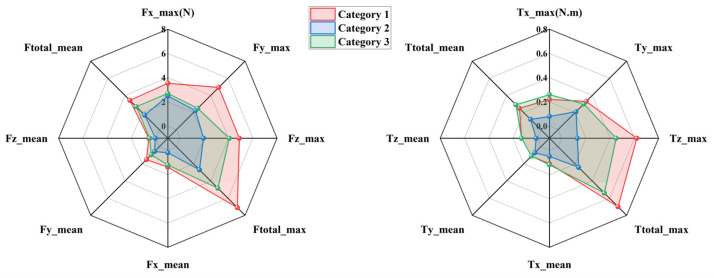
Interaction forces and torques at the upper arm during the eating task.

**Figure 6:**
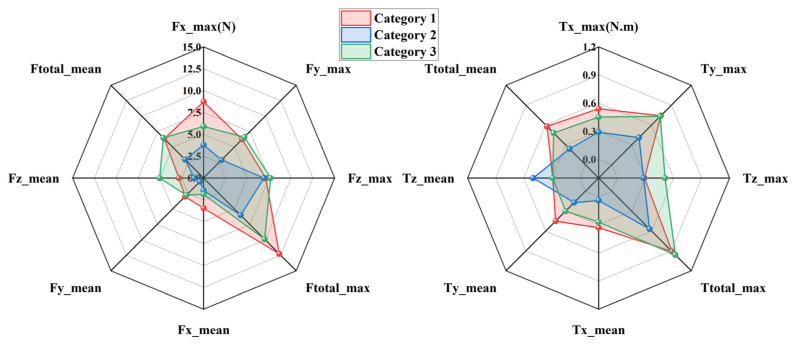
Interaction forces and torques at the forearm during the combing task.

**Figure 7:**
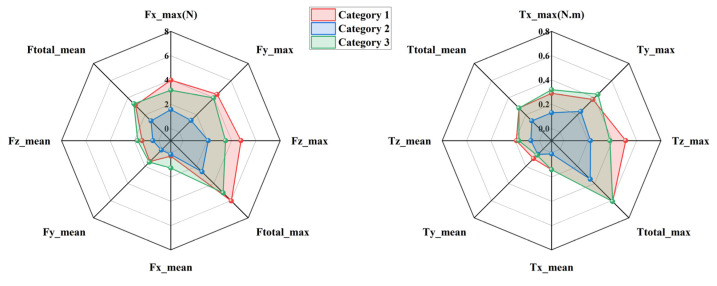
Interaction forces and torques at the upper arm during the combing task.

To evaluate the kinematic compatibility of the three exoskeleton configurations defined in Section 2.1, interaction forces and torques measured at the upper-arm and forearm interfaces were analyzed during two representative rehabilitation tasks, namely eating and combing. The three configurations are distinguished consistently throughout this section, with Category 1 shown in red, Category 2 in blue, and Category 3 in green. For each task and each interface, directional force components and total forces are presented on the left side of Figure 4, Figure 5, Figure 6 and Figure 7, while the corresponding torque components are presented on the right side. Both mean values and peak values are considered in order to characterize steady interaction levels as well as transient loading conditions.

Figure 4 presents the interaction forces and torques measured at the forearm during the eating task. Clear differences can be observed among the three configurations. Category 2 exhibits the smallest overall interaction envelope in both force and torque domains, indicating reduced mechanical constraints at the distal interface. The peak total interaction force of Category 2 remains close to 2.94 N, whereas Category 1 reaches values approaching 7.03 N. Category 3 shows intermediate behavior with peak values around 6.15 N. A similar ordering is observed for the mean total interaction force, confirming that the reduction achieved by Category 2 is not limited to isolated peaks but persists throughout the motion. Directional force components further illustrate the source of these differences. Category 1 shows pronounced forces along the *X* and *Y* directions, suggesting stronger lateral constraints between the forearm and the exoskeleton. In contrast, Category 2 reduces these components by approximately one third, reflecting improved accommodation of transverse misalignment. Forces along the *Z* direction remain relatively small for all configurations, indicating that axial alignment plays a less dominant role in the eating motion. The torque results follow the same trend. Category 2 produces the lowest total interaction torque, with peak values around 0.24 N·m, while Category 1 exceeds 0.7 N·m. The dominant torque component is consistently observed around the *X* axis, which is associated with forearm rotation during eating. The reduced torque levels in Category 2 demonstrate its enhanced ability to absorb rotational mismatch without transmitting excessive loads to the user.

Figure 5 shows the interaction results at the upper-arm interface during the eating task. Compared with the forearm, larger interaction forces are observed for all configurations, reflecting the stronger mechanical coupling at the shoulder. Nevertheless, the relative differences among configurations remain consistent. Category 2 again exhibits the smallest force envelope, with peak total forces around 2.69 N, while Category 1 reaches nearly 7.1 N. Mean force values follow the same ranking, confirming that Category 2 maintains lower interaction levels throughout the task. The most pronounced differences appear in the *Z* direction, where Category 1 generates substantially higher peak forces than the other configurations. This behavior suggests limited capability to compensate for lateral displacement of the glenohumeral joint. Category 2 mitigates these forces effectively, indicating improved self-alignment at the shoulder. In the torque domain, Category 2 consistently produces smaller values across all directions. Peak total torque is reduced by roughly one third compared with Category 1. The dominant contribution arises from rotation about the *Z* axis, which corresponds to shoulder internal and external rotation during feeding. The lower torque levels of Category 2 indicate smoother interaction and reduced constraint during this motion.

Figure 6 illustrates the interaction forces and torques measured at the forearm during the combing task. Compared with the eating task, larger interaction levels are observed due to the increased range of motion and more complex coordination required. Despite this increase, Category 2 consistently maintains the lowest interaction forces. Peak total forces remain near 6 N for Category 2, whereas Category 1 exceeds 12.26 N. Category 3 again lies between the two extremes. Directional analysis reveals that Category 1 exhibits particularly high forces in the *X* and *Y* directions, indicating substantial tangential constraints in the horizontal plane. Category 2 reduces these components noticeably, maintaining values close to 6 N. Torque measurements show a similar pattern. The dominant torque component is associated with forearm rotation, and Category 2 limits the corresponding peak torque to approximately 0.57 N·m. This represents a reduction of nearly 30 percent compared with Category 1. These results indicate that the passive joint distribution of Category 2 remains effective even under more demanding motion conditions.

Figure 7 presents the interaction forces and torques measured at the upper-arm interface during the combing task. Among all tested conditions, this case exhibits the largest interaction levels due to the extensive shoulder motion involved. Category 2 nevertheless maintains the smallest overall force and torque envelopes. Peak total forces for Category 2 are approximately 2.64 N, whereas Category 1 exceeds 6.04 N. The *Y*-direction force is again the dominant contributor, with Category 1 generating markedly higher values than the other configurations. Torque measurements further highlight these differences. Category 1 exhibits pronounced peaks in both *X* and *Z* directions, with total torque values approaching 0.61 N·m. In contrast, Category 2 limits the peak total torque to around 0.35 N·m. This reduction indicates improved accommodation of shoulder rotation and elevation, which are critical during combing. Category 3 consistently shows intermediate behavior, confirming that partial redistribution of passive degrees of freedom provides moderate improvement but does not fully eliminate constraint effects.

Across all tasks and measurement interfaces, a consistent hierarchy of kinematic compatibility is observed. Category 2 exhibits the lowest interaction forces and torques, followed by Category 3, while Category 1 produces the highest interaction levels. Quantitatively, Category 2 achieves reductions of approximately 25 to 40 percent in total interaction forces and up to 40 percent in total torques compared with Category 1. These reductions are observed for both mean and peak metrics, indicating improved behavior during steady motion as well as during transient phases. From a mechanical perspective, the superior performance of Category 2 can be attributed to its more effective distribution of passive degrees of freedom, particularly around the shoulder joint. This distribution enhances self-alignment of the human–exoskeleton closed chain and limits the buildup of residual constraint forces. In contrast, Category 1 exhibits higher sensitivity to joint center mismatch, especially in lateral directions, leading to larger forces and torques. Although all three configurations satisfy theoretical kinematic compatibility conditions, the experimental results demonstrate that their practical compatibility differs substantially. The measurements confirm that appropriate allocation of passive joints is essential for minimizing interaction loads and achieving safe and comfortable upper-limb rehabilitation.

### 3.2. Effect of Passive Joint Release on Interaction Loading

**Figure 8:**
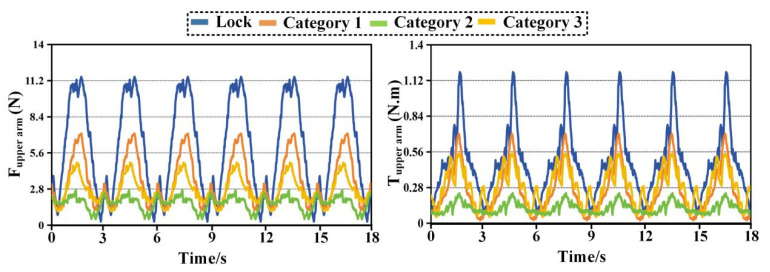
Interaction force at the upper arm during eating with passive joints locked and released.

**Figure 9:**
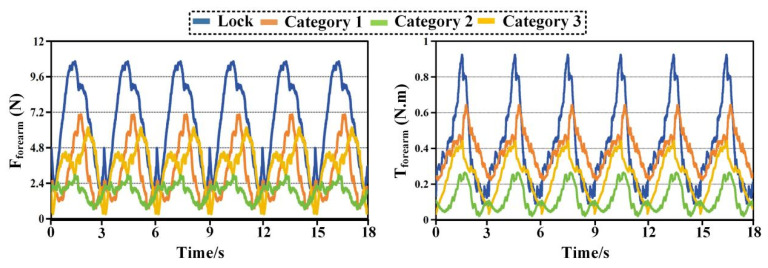
Interaction force at the forearm during eating with passive joints locked and released.

**Figure 10:**
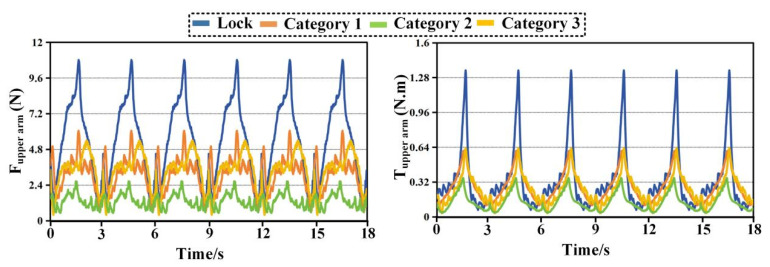
Interaction force at the upper arm during combing with passive joints locked and released.

**Figure 11:**
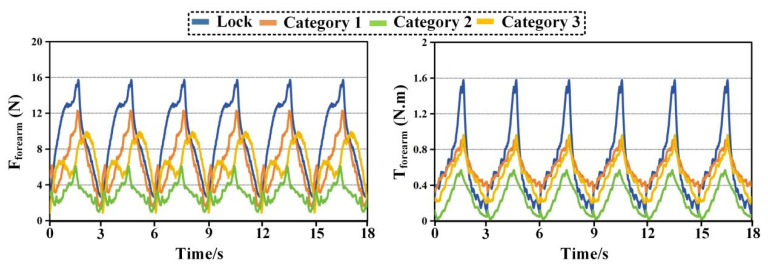
Interaction force at the forearm during combing with passive joints locked and released.

Figure 8, Figure 9, Figure 10 and Figure 11 compare time histories of the resultant interaction force and resultant interaction torque under two operating modes. The curve labeled Lock represents the case where passive joints are locked, while Category 1, Category 2, and Category 3 represent the three configurations with passive joints released. In every plot, the left panel reports interaction force and the right panel reports interaction torque. Across all tasks and both interfaces, locking the passive joints leads to the largest and sharpest periodic peaks, whereas releasing passive joints reduces peak magnitude, lowers the cycle average, and suppresses abrupt transients. Among the three released configurations, Category 2 consistently produces the lowest force and torque levels, indicating the highest compatibility.

In Figure 8, the locked condition shows pronounced periodic peaks in force and torque that repeat over cycles with similar timing, indicating that constraint loads are tightly coupled to the motion phase. The peak force in the locked condition reaches approximately 11.2 N, while the peak torque approaches about 1.2 N·m. When passive joints are released, the interaction envelope shrinks markedly for all three configurations, and the sharp peaks become visibly less aggressive. Category 2 shows the most substantial reduction. Its force peaks remain around 2.5 to 2.8 N, and its torque peaks remain near 0.25 to 0.27 N·m. Relative to the locked condition, this corresponds to a reduction on the order of 70 to 80 percent in both peak force and peak torque. Category 3 also reduces the interaction loads, with peak force around 4.5 to 5 N and peak torque around 0.45 to 0.55 N·m, giving a reduction that is clearly smaller than Category 2 but still substantial. Category 1 exhibits the least improvement among the three released configurations, with peak force roughly 6 to 7 N and peak torque roughly 0.6 to 0.7 N·m. The ordering remains stable throughout the entire time window. Lock remains the highest, Category 1 remains the highest among released cases, Category 2 remains the lowest.

Beyond the peak values, the waveform shape is also informative. The locked condition produces narrow spikes and steep rising edges at similar phases of each cycle, a signature of constraint accumulation followed by rapid release. Releasing passive joints smooths these spikes, suggesting that the passive branch provides motion accommodation before large reaction loads build up. Category 2 provides the smoothest profile and the lowest baseline between peaks, which is consistent with a configuration that best compensates joint axis mismatch at the proximal interface during eating.

Figure 9 shows the same comparison at the forearm interface during eating. The locked condition again yields the largest interaction loads, with force peaks around 9 to 10 N and torque peaks around 0.85 to 0.95 N·m. Releasing passive joints reduces both quantities for all configurations, confirming that the passive branch improves compatibility not only proximally but also distally. Category 2 remains the best performing configuration. Its force peaks are approximately 2.5 to 3 N and its torque peaks are approximately 0.25 to 0.35 N·m. This indicates a peak reduction of roughly 65 to 75 percent compared with the locked condition. Category 3 produces intermediate values with peak force near 4 to 5 N and peak torque near 0.45 to 0.55 N·m. Category 1 remains higher with peak force near 5.5 to 6.5 N and peak torque near 0.6 to 0.7 N·m. The relative spacing among the curves in Figure 9 is slightly smaller than in Figure 8 for the force plot, while the torque plot preserves a clear separation. This difference suggests that at the forearm interface, torque is more sensitive to alignment and rotational accommodation than the net force magnitude during eating. Category 2 shows the strongest reduction in the torque peaks and also reduces the fluctuation amplitude between peaks, implying that it provides the most effective rotational compliance at the distal interface for this task.

Figure 10 reports the combing task at the upper arm interface. The locked condition exhibits peak forces around 10 to 11 N and peak torques around 1.3 to 1.4 N·m. Compared with eating, the torque peaks are noticeably higher, which aligns with the larger shoulder rotation and elevation demanded by combing. Releasing passive joints substantially reduces both force and torque across all configurations. Category 2 again shows the lowest interaction loads. Its peak force remains around 2.5 to 2.7 N and its peak torque remains around 0.30 to 0.34 N·m. The peak reduction relative to the locked case is again close to 65 to 75 percent. Category 3 follows with peak force near 4 to 5 N and peak torque near 0.55 to 0.65 N·m. Category 1 remains higher with peak force near 5 to 6 N and peak torque near 0.55 to 0.65 N·m. A key observation in Figure 10 is the persistence of steep spikes in the locked torque trace at the same phases across cycles. These spikes are strongly attenuated when passive joints are released. This indicates that passive motion compensation is particularly important during combing when shoulder motion is large and joint center mismatch is more likely to translate into constraint torques. Category 2 produces the smallest spikes and the lowest inter-peak baseline, suggesting that it is the most robust configuration for proximal compatibility under demanding shoulder motion.

Figure 11 shows the combing task at the forearm interface and it is the most demanding condition among the four plots in terms of force magnitude. The locked condition reaches force peaks around 15 to 16 N and torque peaks around 1.5 to 1.6 N·m. These values exceed those observed during eating, indicating stronger distal loading during the combing trajectory, likely due to increased lever arm effects and coupled shoulder EL motion. Releasing passive joints reduces both force and torque for all configurations, but the reduction ratios are more differentiated here, making Figure 11 particularly informative for ranking compatibility. Category 2 achieves the largest reduction. Its force peaks decrease to roughly 5 to 6 N, and its torque peaks decrease to roughly 0.55 to 0.60 N·m. This corresponds to an approximate reduction of about 50 percent in peak force and about 60 percent in peak torque relative to the locked condition. Category 3 provides a moderate reduction with peak force around 9 to 10 N and peak torque around 0.85 to 0.95 N·m. Category 1 shows the smallest improvement, with peak force around 11 to 12 N and peak torque around 0.95 to 1.05 N·m. The separation among the three released curves remains consistent across cycles, and Category 2 stays clearly below the others in both force and torque throughout the entire recording. The forearm results during combing highlight an important point. Passive joints do not merely reduce the maximum values, they also reshape the temporal profile. In the locked condition, the steep peaks indicate abrupt constraint accumulation. In the released conditions, the curves become smoother and the peak width increases slightly while the peak height decreases, a typical sign that the mechanism is accommodating misalignment through passive motion rather than storing it as elastic deformation or interface compression. Category 2 exhibits the strongest smoothing effect and the lowest baseline, which provides direct time-domain evidence of better compatibility.

Two conclusions are supported simultaneously by Figure 8, Figure 9, Figure 10 and Figure 11. Releasing passive joints consistently reduces both interaction force and interaction torque at both the upper arm and forearm interfaces for both tasks. This confirms the first-level effect that passive motion compensation improves overall compatibility by alleviating hyperstatic constraints. The second-level comparison shows that the magnitude of reduction depends strongly on configuration. Category 2 yields the largest reductions and the lowest absolute interaction levels across all conditions. Category 3 ranks second, and Category 1 ranks third among the released configurations. The superiority of Category 2 is most evident in the peak values and in the attenuation of sharp spikes, which are the conditions most relevant for comfort and safety during passive rehabilitation.

## 4. Discussion

This study set out to answer a practical question that is often left unresolved in configuration synthesis work. When several exoskeleton configurations satisfy the same theoretical compatibility requirement, do they remain equivalent once a human arm is actually connected and moved through daily living trajectories. The results show they do not. Even under identical hardware, identical trajectories, and the same sensing arrangement, the measured interaction loads differ substantially across configurations. Two observations are especially robust. Releasing passive joints reduces interaction forces and torques at both the upper-arm and forearm interfaces. Among the three compatible configurations, Category 2 yields the lowest interaction loading and the strongest attenuation of the transient peaks, indicating the best practical compatibility.

(1) **Passive joint release reduces hyperstatic loading in a repeatable and task-independent manner**

Across both eating and combing, the locked condition produces the highest interaction forces and torques and it also produces the sharpest periodic peaks. This pattern is important because it suggests that the loads are not random or driven by noise at the interface. They are phase-locked to the motion cycle, which is consistent with constraint accumulation in a closed chain. When passive joints are released, the same trajectories lead to smaller peaks, lower cycle averages, and smoother profiles. The most informative change is the suppression of narrow spikes. A reduction in mean values indicates that the closed chain is, on average, less constrained throughout the movement. A reduction in peak values indicates that the worst phases of the cycle are mitigated, which is directly relevant to comfort and safety.

From a mechanism standpoint, the improvement can be interpreted as a redistribution of mismatch accommodation. With passive joints locked, joint axis and center discrepancies are absorbed primarily through deformation of soft tissues, cuff compliance, and micro-slippage, all of which manifest as abrupt increments in measured force and torque. With passive joints released, a portion of the relative motion is redirected into passive motion of the mechanism, so the mismatch is absorbed as kinematic accommodation rather than interface loading. This is the expected behavior for an even-actuation closed chain in which passive degrees of freedom remove redundant constraints. The experimental evidence supports this interpretation because the reduction appears consistently at both interfaces and for both tasks.

(2) **Category 2 achieves the best practical compatibility among the three compatible configurations**

A key contribution of this work is that the three configurations are not compared solely at the level of geometric plausibility, but are evaluated through measured human–robot interaction loading under representative daily living trajectories. Under identical hardware, trajectories, and sensing arrangements, the three configurations exhibit substantially different interaction force and torque profiles. Among them, Category 2 consistently performs best: its force and torque traces remain below those of Category 1 and Category 3, and this separation is maintained across repeated motion cycles. This indicates that Category 2 does not merely reduce isolated peaks, but establishes a uniformly lower interaction regime throughout the motion.

The distinction among configurations becomes particularly clear when peak behavior is examined. Peaks are the most sensitive indicators of kinematic incompatibility, as they typically arise when multiple joint motions couple and the closed chain momentarily lacks an effective accommodation pathway. Category 2 achieves the greatest reduction in both peak forces and peak torques relative to the locked condition, suggesting that its passive degrees of freedom are positioned and distributed in a way that provides more effective accommodation precisely during phases where mismatch would otherwise concentrate.

Because identical passive joint modules are used across all configurations, the observed differences can be attributed primarily to the configuration-level distribution of passive degrees of freedom rather than to joint-type-dependent mechanical properties. It should be noted that explicit human kinematic modeling based on motion capture systems (e.g., shoulder and EL joint trajectories) was not employed in this study; instead, the matching between passive joint distribution and human kinematics was evaluated indirectly through measured interaction forces and torques during representative functional tasks. Although friction and clearance in practical passive joints may limit ideal motion accommodation even in the released state, these joint-level effects act as common-mode disturbances due to the shared hardware and therefore do not alter the relative performance ranking among configurations.

A plausible mechanical explanation for the superior performance of Category 2 lies in the balance of passive mobility between the shoulder and EL subchains. In a human–exoskeleton closed chain, kinematic mismatch originates at both the shoulder complex and the EL complex. Shoulder center migration and scapular motion introduce time-varying shifts at the proximal end, while soft tissue deformation and forearm rotation coupling introduce secondary misalignment at the distal end. When passive mobility is unevenly distributed, one subchain may dominate the accommodation process, causing residual mismatch to be redirected rather than dissipated. By contrast, a more symmetric allocation of passive mobility reduces the likelihood of such bottlenecks.

This mechanism is reflected in the experimental observations. When the shoulder branch is highly compliant but the EL branch is comparatively constrained, residual mismatch tends to propagate distally and appear as elevated forearm torques. Conversely, when the EL branch is highly compliant but the shoulder branch is constrained, mismatch accumulates proximally and manifests as increased upper-arm forces. Category 2 exhibits consistently reduced interaction loading at both interfaces, indicating a more globally effective accommodation pathway across the closed chain.

(3) **Why the combing task amplifies differences and why this matters**

The combing task typically produces larger interaction torques than eating, especially at the upper arm interface. This observation aligns with the biomechanics of the task. Combing involves larger shoulder elevation and rotation, often requiring coupled motion across multiple anatomical structures and larger center migration at the shoulder complex. In a closed chain, such motions magnify the consequences of any imperfect alignment and any incomplete accommodation. The locked condition shows the strongest spikes under combing, which suggests that combing is a more demanding probe of compatibility than eating. This has direct methodological value. If a configuration performs well under combing, it is likely to be robust under a broader set of daily living motions. The fact that Category 2 maintains the lowest interaction levels under combing strengthens the argument that its compatibility advantage is not limited to a single trajectory. It also suggests a practical screening approach for future designs. A small set of high-demand trajectories can be more discriminative than a large set of low-demand motions when the goal is to rank configurations by compatibility.

(4) **Proximal and distal interfaces provide complementary evidence**

Measuring both upper-arm and forearm interfaces is not redundant. The two interfaces reveal how mismatch and accommodation propagate through the human–robot connection. Proximal loading reflects the ability to accommodate shoulder center migration and multi-axis rotation. Distal loading reflects the ability to accommodate EL axis mismatch, forearm coupling, and the redistribution of residual constraints from the shoulder. In the locked condition, both interfaces exhibit elevated loads, which implies that mismatch is global rather than localized. After passive joints are released, loads decrease at both interfaces but the magnitude of reduction can differ. This difference is informative. A configuration that reduces proximal loads but leaves distal torques relatively high may be compensating shoulder migration at the cost of transferring constraints distally. A configuration that reduces both indicates a more global compatibility improvement. Category 2 exhibits this global improvement pattern, which supports the conclusion that it provides a better overall accommodation pathway across the closed chain.

(5) **Implications for configuration selection and engineering design**

The results support a practical principle for designing compatible upper-limb exoskeletons. Compatibility is not determined only by satisfying a theoretical condition on passive degrees of freedom. It is determined by how those degrees of freedom are distributed relative to the dominant sources of anatomical variability and the dominant directions of motion. Category 2 provides a concrete example of a distribution that performs well across tasks and interfaces. This suggests that symmetric or balanced passive mobility allocation between shoulder and EL subchains can be a strong default choice when the device is intended to support daily living motions with varied shoulder involvement. The time-domain evidence also suggests a second principle. Peak suppression is as important as reducing average loading. Many adverse sensations and safety concerns are triggered by brief high loads rather than sustained moderate loads. Category 2 reduces both peaks and means, while also smoothing the temporal profile. From an engineering perspective, this indicates that the passive branch is not merely acting as a compliance element but is functioning as a kinematic accommodation mechanism that prevents abrupt constraint buildup.

In addition, the observed role of the base-connected prismatic joints is consistent with their intended function. Transferring device weight to the base reduces gravitational contributions to interface loads, which helps ensure that measured interaction forces are dominated by kinematic mismatch rather than by static weight support. This is essential when the goal is to attribute differences primarily to configuration effects.

(6) **Clinical and experimental relevance**

Although the experiments were performed with healthy participants in passive mode, the implications are relevant to clinical rehabilitation. Passive training is often used early after stroke when voluntary control is limited. In this phase, comfort and safety are particularly sensitive to high interaction peaks. A configuration that reduces peak interaction forces and torques can reduce the likelihood of discomfort, improve tolerance to training, and potentially allow longer sessions or higher repetition counts. The consistency of Category 2 across tasks suggests that it may offer a more forgiving mechanical interface for heterogeneous users, including those with altered muscle tone or limited range of motion. The locked condition results are also useful beyond being a baseline. They highlight what happens when accommodation is removed. This provides a cautionary reference for designs that rely on tight alignment assumptions. Even when careful initial alignment is performed, time-varying mismatch during motion can still generate large loads if the mechanism has insufficient passive accommodation.

(7) **Limitations**

Several limitations should be considered when interpreting the results. First, the study used a small sample size and healthy participants. While the closed-chain mechanics and the accommodation mechanism should generalize, patient populations may exhibit different soft tissue compliance, altered joint kinematics, and different tolerance to interface pressure. Second, the experiments were conducted in passive trajectory tracking. Active participation, spasticity, or involuntary movements may introduce additional interaction components that interact with the passive branch differently. Third, the analysis emphasized resultant forces and torques and summary metrics. While these metrics are appropriate for configuration ranking, additional insight could be gained by relating specific phases of the trajectories to anatomical events such as shoulder elevation onset or EL flexion peaks.

Another limitation is that the current results focus on net interaction loads rather than relative motion offsets at the cuffs. Force reduction strongly suggests improved accommodation, but a combined analysis of force and relative slip would provide a more complete picture of interface mechanics and could help identify whether reductions are achieved through kinematic accommodation or through controlled micro-slippage. Finally, only two daily living trajectories were used. They are representative and discriminative, especially combing, but broader coverage would further test generality.

(8) **Future work**

Several extensions of the present study follow naturally. A first direction is to compute reduction ratios systematically for each metric, each interface, and each task. Presenting these ratios in a compact table would make the ranking of configurations more transparent and would enable formal statistical testing of performance differences. A second direction is to incorporate participant-specific alignment offsets and anthropometric variability into the analysis in order to examine robustness. Although Category 2 appears robust across the tested tasks, a variability-focused study could assess whether this advantage is preserved across a wider range of body sizes and joint center migration patterns. A third direction is to bridge the experimental findings to design optimization. While the current study compares three configuration families derived from synthesis, future work can treat passive joint placement and passive joint range limits as explicit design variables and optimize them for minimizing peak interaction loads under a representative set of trajectories. In this context, the systematic optimization of passive joint stroke limits under broader anthropometric variability and more extreme motion scenarios is of particular interest. Furthermore, incorporating explicit human kinematic modeling would enable a quantitative analysis of the matching between passive joint placement and human joint motion, thereby supporting the optimization of passive joint spatial positions in addition to their quantity and distribution. Mechanical and control-level strategies to further reduce passive joint friction and clearance effects, such as low-friction joint structures or compensation methods, can also be integrated into this optimization framework. Finally, extending the experimental protocol to clinical users and to active assistive rehabilitation modes represents an important step toward translation. In such settings, voluntary muscle activation and assistive control may interact with passive joint accommodation, potentially altering the interaction dynamics observed in passive training. Investigating whether the stiffness or damping characteristics of passive joints should be adapted for active assistive modes is therefore an important topic for future research. In these clinically relevant scenarios, improvements in compatibility may directly influence training adherence, comfort, and functional outcomes.

## 5. Conclusions

This study investigated the practical kinematic compatibility of three theoretically compatible upper limb exoskeleton configurations derived from the synthesis framework in Li et al. Interaction forces and torques were measured at both the upper-arm and forearm interfaces during two representative passive rehabilitation motions, namely eating and combing. Two operating modes were evaluated for each configuration, one with passive joints released and one with passive joints locked, enabling direct assessment of the contribution of passive motion accommodation. The experimental results lead to three main conclusions. First, releasing the passive joints consistently reduced interaction forces and torques across both tasks and both interfaces. This confirms that passive degrees of freedom provide effective motion accommodation in the human–exoskeleton closed chain and alleviate hyperstatic loading that would otherwise arise from joint center mismatch. Second, the magnitude of improvement depended strongly on configuration, even though all three configurations satisfy theoretical compatibility conditions. Category 2 achieved the lowest interaction loading and the strongest suppression of transient peaks in both force and torque, indicating the best practical compatibility. Category 3 showed intermediate performance, while Category 1 produced the highest interaction levels among the released configurations. Third, the combing task amplified configuration-dependent differences more than the eating task, highlighting its value as a discriminative trajectory for compatibility assessment under demanding shoulder motion.

Overall, these findings demonstrate that compatibility evaluation must go beyond theoretical DOF criteria and consider measured interaction loading under representative motions. The proposed experimental protocol and the resulting evidence provide guidance for configuration selection in upper-limb rehabilitation exoskeleton design, supporting the use of configuration Category 2 when the goal is to minimize interaction forces and improve comfort and safety during passive training.

## Figures and Tables

**Table 1:** Three types of 4-DOF exoskeleton configurations compatible with the upper limb.

Sub-Chain Category	Number of Active Joints	Number of Passive Joints		
		Category 1	Category 2	Category 3
GH joint sub-chain	3	*f_s_* = 3	*f_s_* = 4	*f_s_* = 5
EL joint sub-chain	1	*f_e_* = 5	*f_e_* = 4	*f_e_* = 4

**Table 2:** The technical specifications of the sensors and data acquisition hardware.

Torque Sentor		Data Acquisition	
Specification	Details	Specification	Details
Model	Mini45-SI-145-5	Model	ATI Net F/T DAQ System (ATI Industrial Automation, Apex, NC, USA)
Measurement Range	F_x_,F_y_ (±145 N); F_z_ (±290 N); T_x_,T_y_,T_z_ (±5 N·m)	Sampling Rate	7000 samples/second per channel
Uncertainty	±0.5% of full scale	Uncertainty	±0.01% of reading
Bandwidth	1000 Hz	Resolution	16-bit
Resolution	0.01 N; 0.001 N·m	Input Range	±10 V
Nonlinearity	<±0.2% of full scale	Communication Interface	Ethernet, USB, Serial

## Data Availability

The data presented in this study are available on request from the corresponding author.
